# Supplementary material for: The mark of vegetation change on Earth’s surface energy balance
Source: Nat Commun. 2018 Feb 20;9:679. doi: 10.1038/s41467-017-02810-8 (PMC5820346; doi:10.1038/s41467-017-02810-8)
Supplement: Supplementary file 1 — Supplementary Information [file 41467_2017_2810_MOESM1_ESM.pdf]

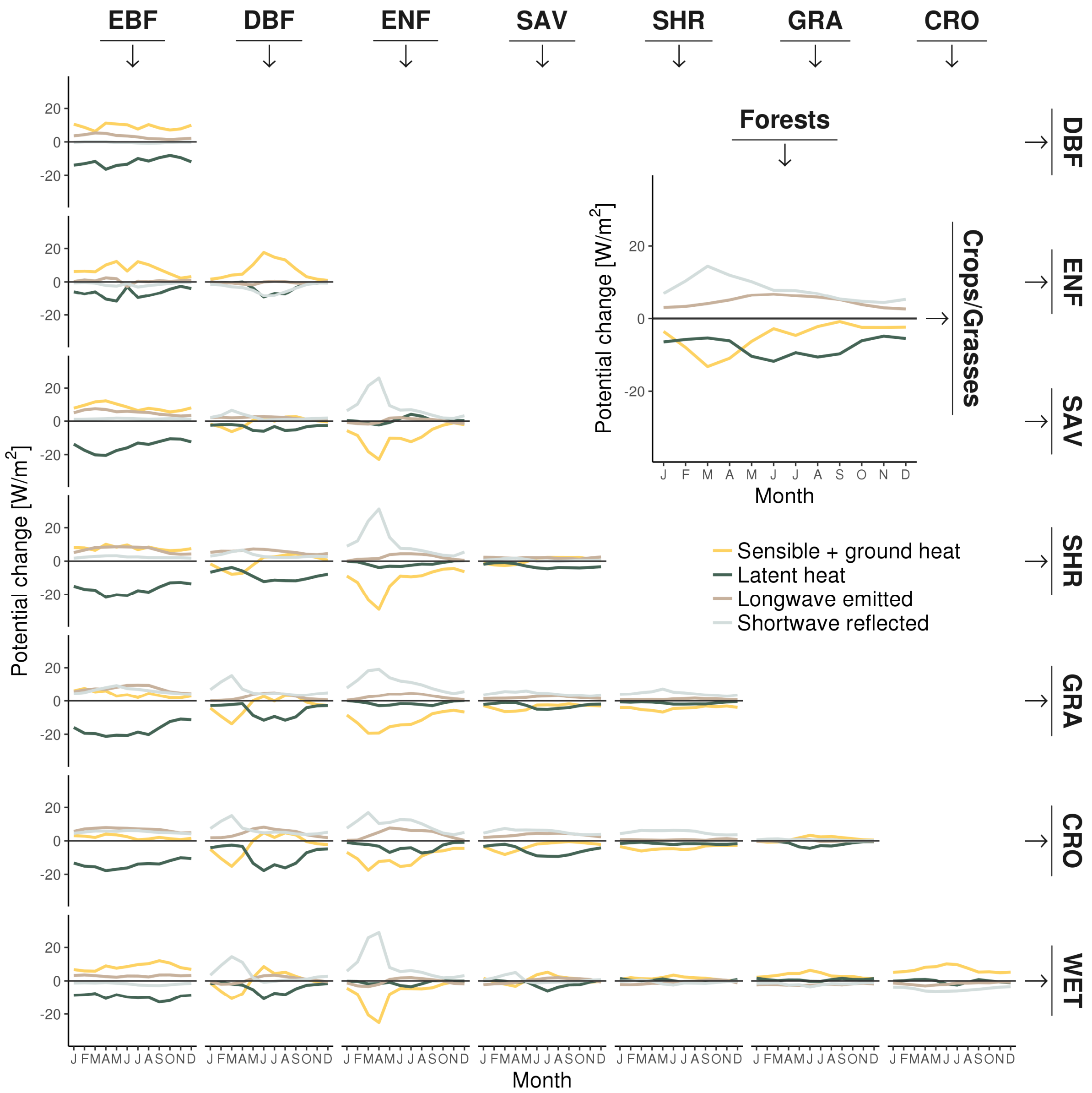

Supplementary Figure 1 | Global summary of the seasonal potential change in surface energy balance for various transitions in vegetation type as derived from satellite observations. The transitions shown involve the following vegetation classes: evergreen broadleaf forests (EBF), deciduous broadleaf forests (DBF), evergreen needleleaf forests (ENF), savannas (SAV), shrublands (SHR), grasslands (GRA), croplands (CRO) and wetlands (WET). Because transitions are symmetric, reverse transitions can be derived by inverting the sign. The inset shows a more generic transition from Trees to either Crops or Grasses corresponding to the maps shown in Figures 1 and 2 of the main text. Letters in the x-axis represents the months of the year.

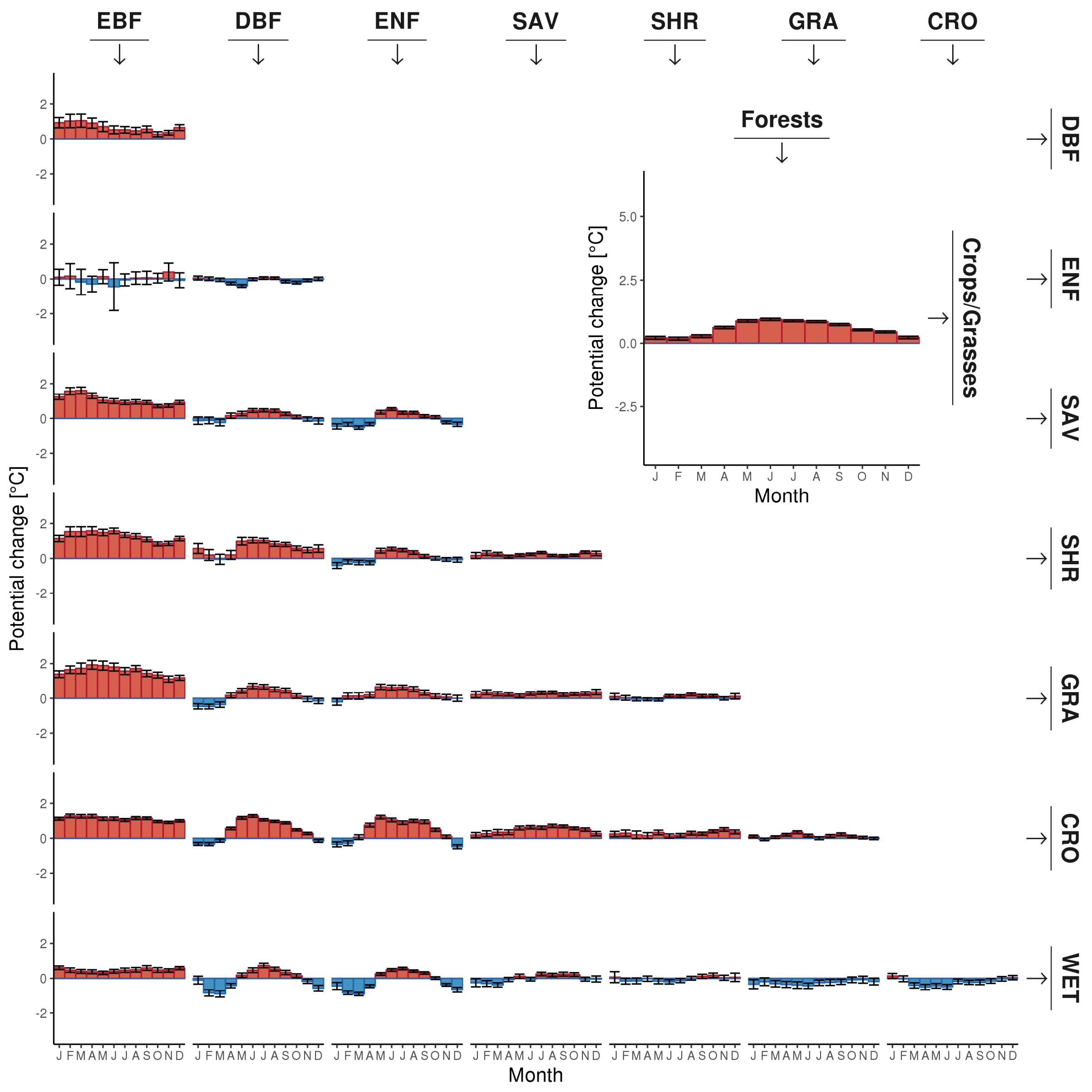

Supplementary Figure 2 | Global summary of the seasonal potential change in mean land surface temperature for various transitions in vegetation type as derived from satellite observations. For descriptions of the vegetation classes, see Supplementary Figure 1. Letters in the x-axis represents the months of the year. The confidence interval represents plus or minus two times the standard error around the mean.

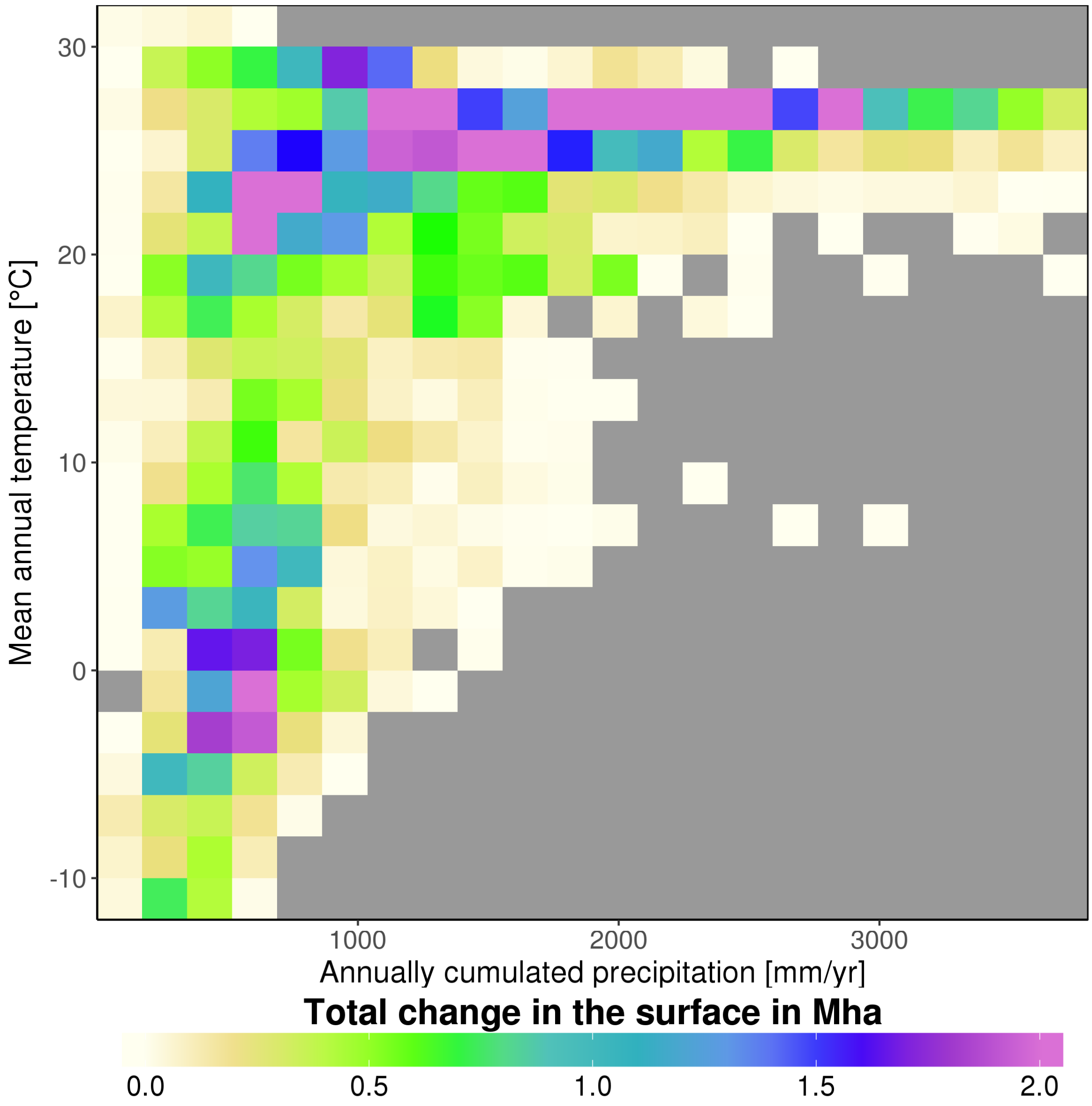

Supplementary Figure 3 | Total change in area from 2000 to 2015 under different climate gradients. Change relates to all vegetation transitions considered in this study together, according to the ESA CCI land cover maps[1]. The climate axes are calculated based on CRU data v4.00 at 0.5° × 0.5° resolution.

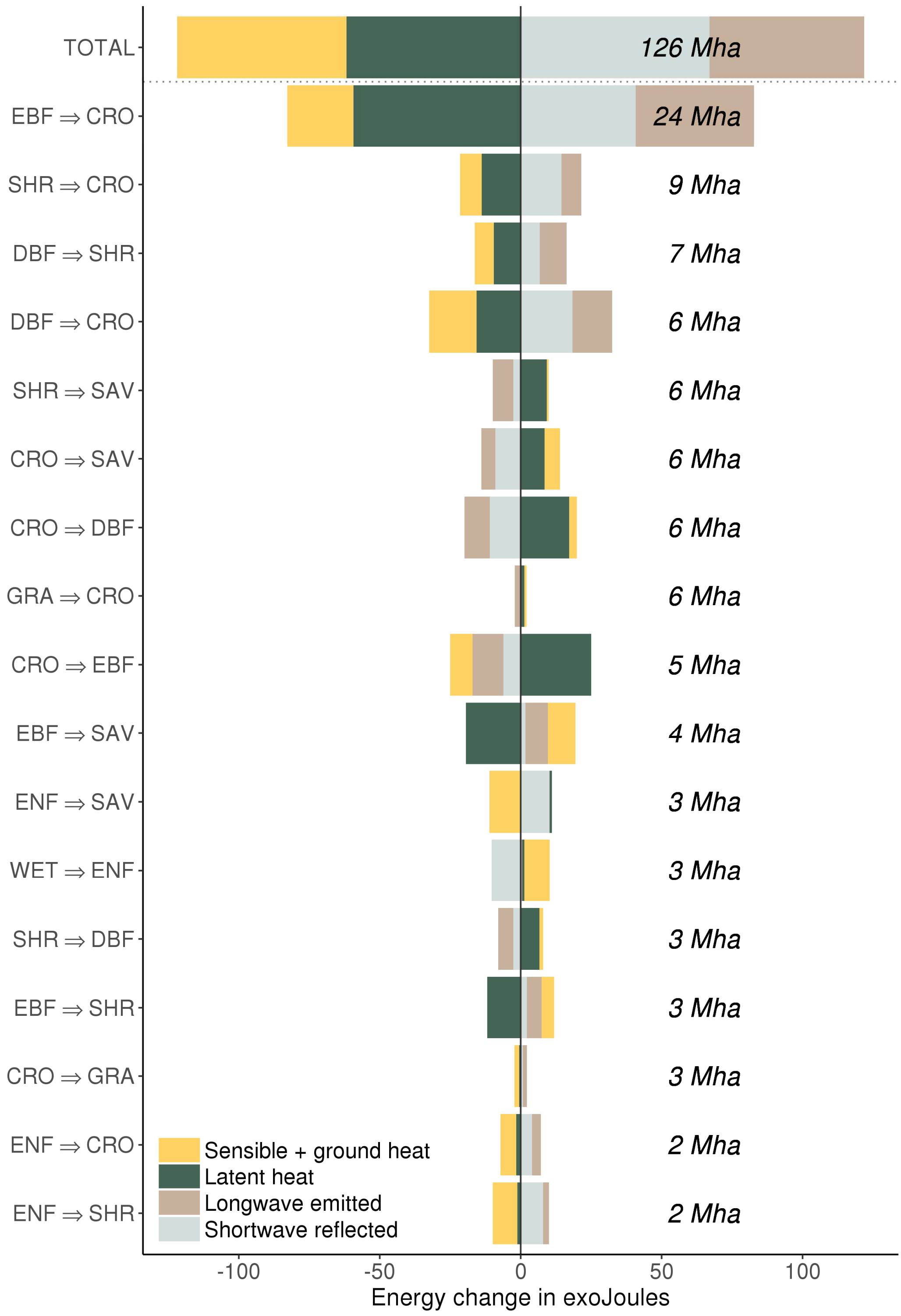

Supplementary Figure 4 | Cumulated changes in energy for each component of the surface energy balance resulting from major vegetation transitions that occurred between 2000 and 2015. Transitions are sorted according to decreasing changed area. The changed area per transition, calculated based on the ESA CCI land cover maps[1], are reported in megahectares on the right of the bars. The transitions shown involve the following vegetation classes: evergreen broadleaf forests (EBF), deciduous broadleaf forests (DBF), evergreen needleleaf forests (ENF), savannas (SAV), shrublands (SHR), grasslands (GRA), croplands (CRO) and wetlands (WET).

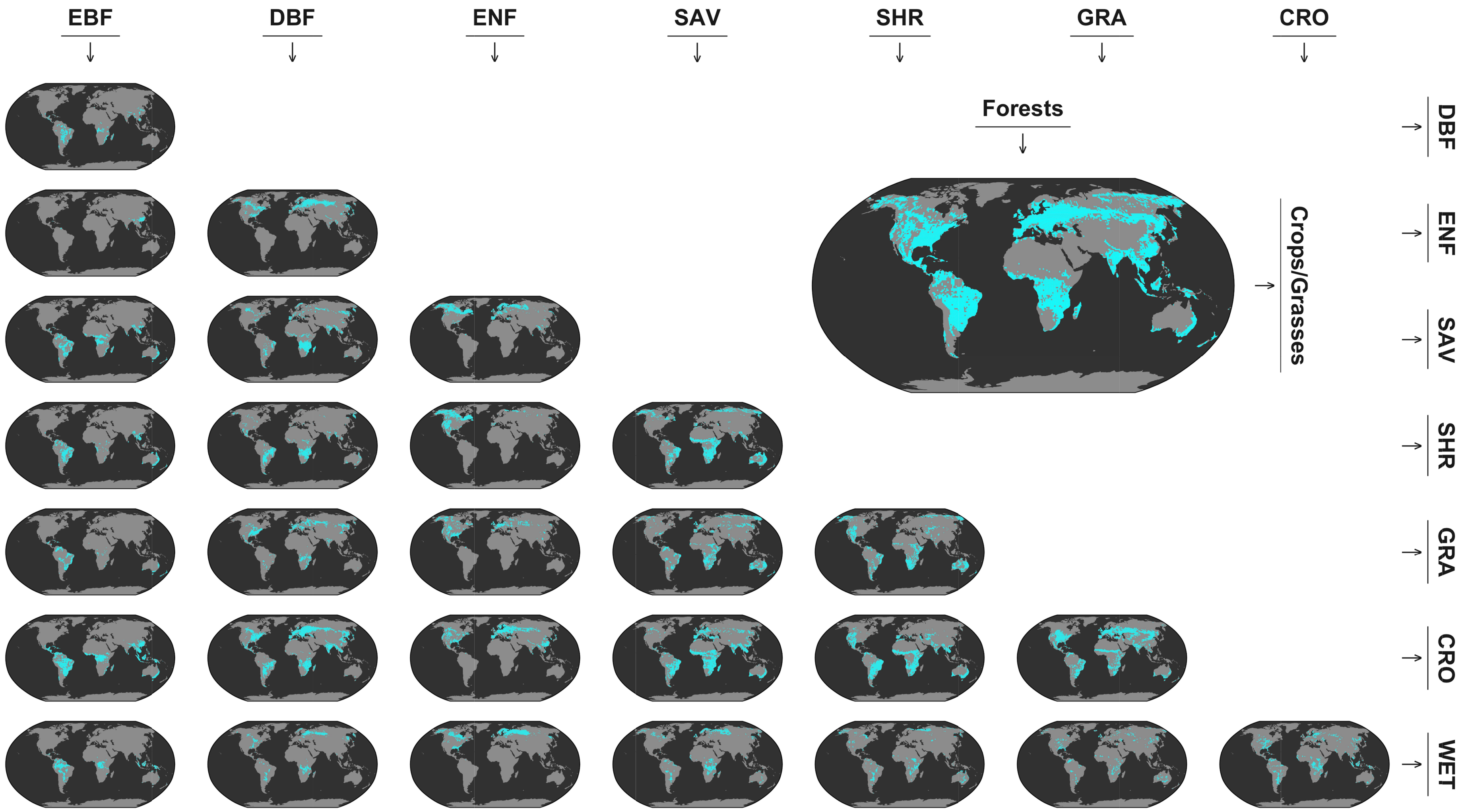

Supplementary Figure 5 | Areas where data is available in the final dataset for different vegetation transitions. For descriptions of the vegetation classes, see Supplementary Figure 1.

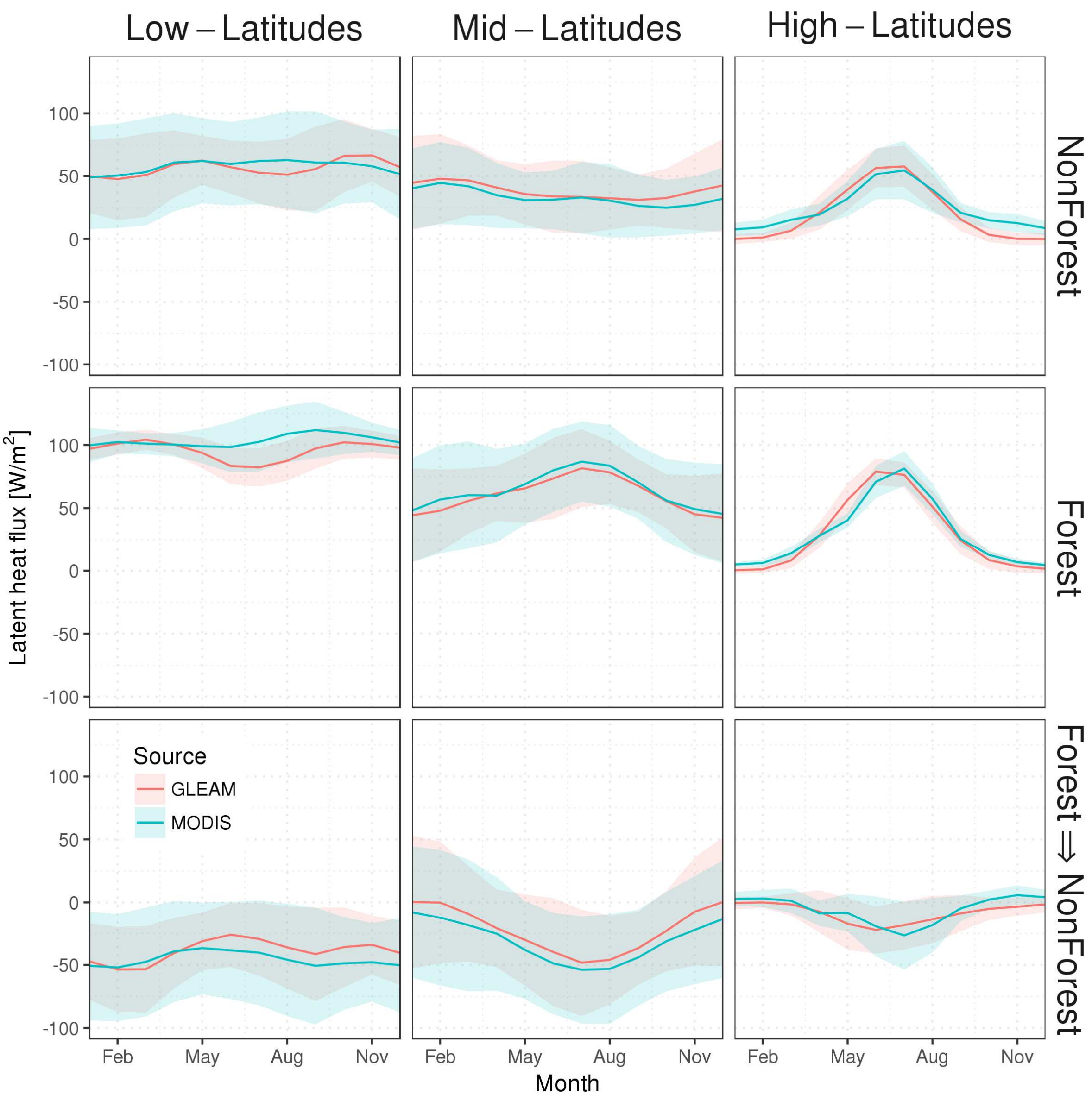

Supplementary Figure 6 | Inter-comparison of the latent heat flux products. The MODIS MOD16A2 product[2] used in the main paper is compared against the GLEAM v3.1 product [3,4] for all pixels at 1°spatial resolution with high forest cover (>75%) against those with low forest cover (<25%). Forest cover is derived from the ESA CCI land cover map[1] of 2010.

Supplementary Table 1 | Cross-walking table to translate land cover to vegetation classes. Using a dedicated approach[6], land cover classes used in the ESA CCI maps[1] are converted to the following vegetation classes: evergreen broadleaf forests (EBF), deciduous broadleaf forests (DBF), evergreen needleleaf forests (ENF), deciduous needleleaf forests (DNF), mixed forests (MF), savannas (SAV), shrublands (SHR), grasslands (GRA), croplands (CRO), wetlands (WET), urban (URB), water (WAT), permanent snow/ice (SNO) and bare/sparsely vegetation (BSV). The value in each cell indicates the percentage of attribution from a 300m land cover class to a 0.05° vegetation class. Vegetation cover classes are also regrouped in broader classes according to the colours.

FAO Land Cover Classification System[5]		*IGBP classification*													
**Code**	**Description**	**EBF**	**DBF**	**ENF**	**DNF**	**MF**	**SAV**	**SHR**	**GRA**	**CRO**	**WET**	**URB**	**WAT**	**SNO**	**BSV**
10	Cropland, rainfed	-	-	-	-	-	-	-	-	100	-	-	-	-	-
11	Herbaceous cover	-	-	-	-	-	-	-	-	100	-	-	-	-	-
12	Tree or shrub cover	-	-	-	-	-	-	-	-	100	-	-	-	-	-
20	Cropland, irrigated or post-flooding	-	-	-	-	-	-	-	-	100	-	-	-	-	-
30	Mosaic cropland (>50%) / natural vegetation (tree, shrub, herbaceous cover) (<50%)	-	-	-	-	-	-	-	-	100	-	-	-	-	-
40	Mosaic natural vegetation (tree, shrub, herbaceous cover) (>50%) / cropland (<50%)	-	-	-	-	-	-	-	-	100	-	-	-	-	-
50	Tree cover, broadleaved, evergreen, closed to open (>15%)	100		-	-	-	-	-	-	-	-	-	-	-	-
60	Tree cover, broadleaved, deciduous, closed to open (>15%)	-	100	-	-	-	-	-	-	-	-	-	-	-	-
61	Tree cover, broadleaved, deciduous, closed (>40%)	-	100	-	-	-	-	-	-	-	-	-	-	-	-
62	Tree cover, broadleaved, deciduous, open (15-40%)	-	-	-	-	-	100	-	-	-	-	-	-	-	-
70	Tree cover, needleleaved, evergreen, closed to open (>15%)	-	-	100	-	-	-	-	-	-	-	-	-	-	-
71	Tree cover, needleleaved, evergreen, closed (>40%)	-	-	100	-	-	-	-	-	-	-	-	-	-	-
72	Tree cover, needleleaved, evergreen, open (15-40%)	-	-	-	-	-	100	-	-	-	-	-	-	-	-
80	Tree cover, needleleaved, deciduous, closed to open (>15%)	-	-	-	100	-	-	-	-	-	-	-	-	-	-
81	Tree cover, needleleaved, deciduous, closed (>40%)	-	-	-	100	-	-	-	-	-	-	-	-	-	-
82	Tree cover, needleleaved, deciduous, open (15-40%)	-	-	-	-	-	100	-	-	-	-	-	-	-	-
90	Tree cover, mixed leaf type (broadleaved and needleleaved)	-	-	-	-	100	-	-	-	-	-	-	-	-	-
100	Mosaic tree and shrub (>50%) / herbaceous cover (<50%)	-	-	-	-	-	100	-	-	-	-	-	-	-	-
110	Mosaic herbaceous cover (>50%) / tree and shrub (<50%)	-	-	-	-	-	100	-	-	-	-	-	-	-	-
120	Shrubland	-	-	-	-	-	-	100	-	-	-	-	-	-	-
121	Shrubland evergreen	-	-	-	-	-	-	100	-	-	-	-	-	-	-
122	Shrubland deciduous	-	-	-	-	-	-	100	-	-	-	-	-	-	-
130	Grassland	-	-	-	-	-	-	-	100	-	-	-	-	-	-
140	Lichens and mosses	-	-	-	-	-	-	-	-	-	-	-	-	-	100
150	Sparse vegetation (tree, shrub, herbaceous cover) (<15%)	-	-	-	-	-	-	-	-	-	-	-	-	-	100
152	Sparse shrub (<15%)	-	-	-	-	-	-	-	-	-	-	-	-	-	100
153	Sparse herbaceous cover (<15%)	-	-	-	-	-	-	-	-	-	-	-	-	-	100
160	Tree cover, flooded, fresh or brakish water	-	-	-	-	-	-	-	-	-	100	-	-	-	-
170	Tree cover, flooded, saline water	-	-	-	-	-	-	-	-	-	100	-	-	-	-
180	Shrub or herbaceous cover, flooded, fresh/saline/brakish water	-	-	-	-	-	-	-	-	-	100	-	-	-	-
190	Urban areas	-	-	-	-	-	-	-	-	-	-	100	-	-	-
200	Bare areas	-	-	-	-	-	-	-	-	-	-	-	-	-	100
201	Consolidated bare areas	-	-	-	-	-	-	-	-	-	-	-	-	-	100
202	Unconsolidated bare areas	-	-	-	-	-	-	-	-	-	-	-	-	-	100
210	Water bodies	-	-	-	-	-	-	-	-	-	-	-	100	-	-
220	Permanent snow and ice	-	-	-	-	-	-	-	-	-	-	-	-	100	-

References

1. ESA. *Land Cover CCI Product User Guide Version 2*. (2017). at <http://maps.elie.ucl.ac.be/CCI/viewer/download/ESACCI-LC-Ph2-PUGv2_2.0.pdf>

2. Mu, Q., Heinsch, F., Zhao, M. & Running, S. W. Development of a global evapotranspiration algorithm based on MODIS and global meteorology data. *Remote Sens. Environ.* **106,** 285–304 (2007).

3. Miralles, D. G. *et al.* Global land-surface evaporation estimated from satellite-based observations. *Hydrol. Earth Syst. Sci.* **15,** 453–469 (2011).

4. Martens, B. *et al.* GLEAM v3: Satellite-based land evaporation and root-zone soil moisture. *Geosci. Model Dev.* **10,** 1903–1925 (2017).

5. Di Gregorio, A. *Land CoverClassification System (LCCS). Classification concepts and user manual. Software version 2.* (2005). at <http://www.fao.org/docrep/008/y7220e/y7220e00.HTM>

6. Poulter, B. *et al.* Plant functional type classification for Earth System Models: results from the European Space Agency's Land Cover Climate Change Initiative. *Geosci. Model Dev.* **8,** 2315–2328 (2015).
